# Cannabinoid receptor ligands modulate fibrosis and inflammation in idiopathic pulmonary fibrosis: a preliminary study

**DOI:** 10.55730/1300-0152.2713

**Published:** 2024-10-23

**Authors:** Sevil KÖSE, Selin ÖNEN, Merve GİZER, Esin BODUROĞLU, Uğur GÖNÜLLÜ, Petek KORKUSUZ

**Affiliations:** 1 Department of Plastic, Reconstructive and Aesthetic Surgery, Faculty of Medicine, Akdeniz University, Antalya, Turkiye; 2 Department of Medical Biology, Faculty of Medicine, Atılım University, Ankara, Turkiye; 3 METU MEMS Center, Ankara, Turkiye; 4 Department of Pathology, Medical School, Atılım University, Ankara, Turkiye; 5 Department of Pulmonary Diseases, Medical School, Atılım University, Ankara, Turkiye; 6 Department of Histology and Embryology, Faculty of Medicine, Hacettepe University, Ankara, Turkiye

**Keywords:** Idiopathic pulmonary fibrosis, cannabinoid, cannabinoid receptor, inflammation, fibrosis

## Abstract

**Background/aim:**

No specific pharmacological treatment regimen for idiopathic pulmonary fibrosis (IPF) exists. Therefore, new antiinflammatory therapeutic strategies are needed. Cannabinoids (CBs), known for their inflammation-modulating and antifibrotic effects, may be potential medication candidates for treating IPF. We aim to evaluate the inflammation-modulating and antifibrotic effects of CB receptor (CBR) agonists and antagonists in lipopolysaccharide-stimulated normal human lung fibroblast, epithelial cells, IPF fibroblast cells, and monocytes.

**Materials and methods:**

We detected CBRs in normal human lung fibroblasts (LL24) and IPF fibroblast cells (LL29), epithelial cells (A549) and monocytes (THP-1) by flow cytometry. We determined TGF-β1, IL-8, and TNF-α inflammatory cytokines in the LL24, LL29, A549, and THP-1 cell culture supernatants on days 1 and 5 by ELISA. We evaluated the cell viability in LL24, LL29, and A549 cells on days 1, 3, and 5 spectrophotometrically and detected collagen Type I (ColI) production in the LL24 and LL29 cell culture supernatants on days 1, 3, and 5 by ELISA.

**Results:**

LL24, LL29, A549, and THP-1 cells exhibited CB1 (CB1R) and CB2 (CB2R) receptors. CB1R and CB2R agonists WIN55,212-2 and JWH015 inhibited fibroblastic and epithelial cell proliferation on day 5. TGF-β1 and TNF-α release increased, while IL-8 release decreased in LL24, LL29, A549, and THP-1 cells in response to the administration of WIN55,212-2 and JWH015 at a 10^−2^ mM concentration. CB1R and CB2R antagonists AM251 and AM630 did not block agonistic responses, suggesting a nonclassical CBR-mediated pathway. CB2R agonist JWH015 decreased ColI expression in IPF lung fibroblasts LL29 on day 3.

**Conclusion:**

These results suggest that CB signaling regulates the progression of pulmonary inflammation and fibrosis via CBR activation. This may offer a potential pharmacological tool for developing antifibrosis therapies.

## 1. Introduction

Idiopathic pulmonary fibrosis (IPF) is a progressive and ultimately fatal disease characterized by progressive fibrosis (Raghu, 2017). Epidemiological studies point to a prevalence of 10–60 cases per 100,000 individuals, and this rate has increased after the COVID-19 pandemic (Raimundo et al., 2016). Although its etiology is unknown, lung fibrosis in IPF results from increased tissue repair and remodeling, including chronic inflammation and recurrent epithelial regeneration (Wynn, 2011). Clinical trials using antiinflammatory and antifibrotic drugs such as nintedanib and prednisone (Maher and Strek, 2019; Somogyi et al., 2019) have been conducted to achieve a complete clinical cure of IPF (Raghu, 2017). Although these new therapies have led to better prognosis and delayed disease progression, IPF remains an incurable disease (Raimundo et al., 2016; Heukels et al., 2019). In addition, nintedanib and prednisone have side effects such as gastrointestinal toxicity (Cottin, 2017; Lancaster et al., 2017), drug-induced liver injury (Verma et al., 2018), risk of bleeding (Corte et al., 2015), and arterial thromboembolism (Corte et al., 2015). As of March 2024, 406 studies are ongoing within the scope of ‘IPF’ and ‘treatment’ according to the ClinicalTrials.gov database; these studies have focused on new therapeutic protocols with existing drugs or the use of active substances with known antifibrotic and/or antiinflammatory effects, such as cannabinoids (CBs), for treatment of IPF (Heukels et al., 2019; Liu et al., 2022).

CB1 (CB1R) (Gkoumassi et al., 2007; Servettaz et al., 2010; Karmaus et al., 2013; Liu et al., 2014a; Bronova et al., 2015; Staiano et al., 2016; Cinar et al., 2017; Muthumalage and Rahman, 2019; Mohammed et al., 2020; Zawatsky et al., 2020; Chen et al., 2022) and CB2 (CB2R) (Gkoumassi et al., 2007; Servettaz et al., 2010; Costola-de-Souza et al., 2013; Karmaus et al., 2013; Staiano et al., 2016; Bort et al., 2017; Liu and Shi, 2019; Muthumalage and Rahman, 2019; Liu et al., 2020; Parlar et al., 2021; Liu et al., 2022) receptor agonists exhibit inflammation-modulating (Gkoumassi et al., 2007; Costola-de-Souza et al., 2013; Karmaus et al., 2013; Liu et al., 2014b; Staiano et al., 2016; Bort et al., 2017; Cinar et al., 2017; Liu and Shi, 2019; Muthumalage and Rahman, 2019; Liu et al., 2020; Mohammed et al., 2020; Zawatsky et al., 2020; Parlar et al., 2021; Liu et al., 2022) and antifibrotic effects (Servettaz et al., 2010; Bronova et al., 2015; Liu and Shi, 2019; Chen et al., 2022) in lung tissue or bronchoalveolar lavage fluid (BALF) by focusing on inflammation markers, including transforming growth factor-β1 (TGF-β1), interleukin-8 (IL-8), and tumor necrosis factor-α (TNF-α) and collagen type I (ColI) in human bronchial epithelial cells (Gkoumassi et al., 2007; Muthumalage and Rahman, 2019), human embryonic lung fibroblasts (Liu and Shi, 2019; Muthumalage and Rahman, 2019), and monocyte and lung resident macrophages (Staiano et al., 2016; Muthumalage and Rahman, 2019). In addition, human bronchial epithelial cells (Gkoumassi et al., 2007; Boyacioglu et al., 2021), lung macrophages (Staiano et al., 2016), and mice lung tissue (Tahamtan et al., 2018; Huang et al., 2020) express CB receptors (CBRs).

In this study, we hypothesized that CBs, known for their antifibrotic and inflammation-modulating effects, and CBRs distributed in lung tissue might effectively treat IPF. Our first objective is to assess CB1R and CB2R expression on LL24, LL29, A549, and THP-1 cells. The second objective is to analyze the effect of CB1R and CB2R agonists WIN55-212,2 and JWH015 on TGF-β1, IL-8, and TNF-α inflammatory cytokine release from LL24, LL29, A549, and THP-1 cells in a dose-dependent manner by ELISA. The third objective is to evaluate the antifibrotic effect of WIN55-212,2 and JWH015 in a dose- and time-dependent manner by observing the viability and ColI release of LL24, LL29, and A549 cells using MTT viability assay and ELISA, respectively, and set the half-maximal inhibitory concentration (IC_50_) dose.

## 2. Materials and methods

### 2.1. Study design

**Figure 1:**
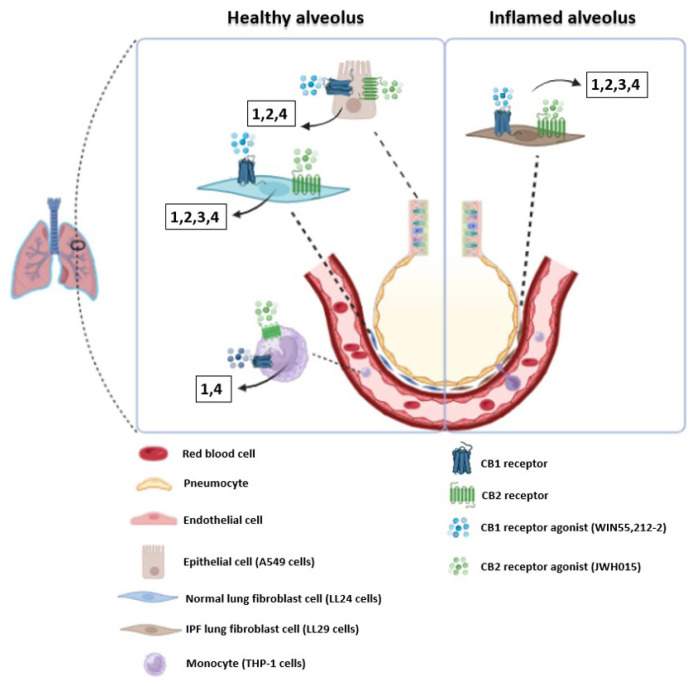
Schematic design of the study. We analyzed the presence of CB1R and CB2R (1), cell viability (2), release of ColI (3), and inflammation markers (TNF-α, IL-8, and TGF-β) (4) in LPS+ normal human lung fibroblast cells (LL24), human lung IPF fibroblast cells (LL29), human lung epithelial cells (A549), and human PB monocytes (THP-1).

We designed a prospective, randomized, and controlled in vitro study. Independent variables are time (1, 3, and 5 days) and groups [LPS stimulated (LPS+) and nonstimulated (LPS–) normal human lung fibroblasts (LL24) and IPF fibroblast cells (LL29), lung epithelial cells (A549), and peripheral blood monocytes (THP-1)]. Dependent variables are CBR expressions, quantitative measurements of inflammation markers (TGF-β1, IL-8, and TNF-α), ColI, and cellular metabolic activity. This in vitro study used commercially available cell lines, and no human or animal subjects were involved. Therefore, ethics committee approval was not required. However, all experimental procedures were conducted in accordance with ethical standards and guidelines to ensure the integrity and ethical compliance of the research. We determined biological replicates with power analysis (G-Power v3.1). Figure 1 illustrates the experimental setup schematically.

### 2.2. Drugs

We purchased CB1R full agonist WIN55,212-2 (#W102), CB2R full agonist JWH015 (#504274), CB1R full antagonist AM251 (#A6226), CB2R full antagonist AM630 (#SML0327), and LPS (#L2630) from Sigma-Aldrich (St Louis, MO, USA).

### 2.3. Cell culture

We obtained LL29 (#CCL-134), LL24 (#CCL-151), A549 (#CCL-185), and THP-1 (#TIB-202) cell lines from American Type Culture Collection (ATCC, Manassas, VA, USA). LL24, LL29, and A549 cells were cultured in Kaighn’s Modification of F-12K Medium (ATCC, #30-2004) containing 10% FBS (PAN Biotech, #P30-193306, Aidenbach, Germany). THP-1 cells were cultured in RPMI-1640 (ATCC, #30-2001) and contained 10% FBS and 0.05 mM 2-mercaptoethanol (Sigma-Aldrich, #M6250). All cells were incubated with 5% CO_2_ at 37 °C.

### 2.4. Flow cytometry

CB1R and CB2R expressions were measured in LL24, LL29, A549, and THP-1 cells by FC. The cells were permeabilized using 0.2% Tween20 (Abcam, #ab128987, Cambridge, UK) before labeling. We performed an indirect immunofluorescence assay using rabbit antihuman CB1 (#ab3558), rabbit antihuman CB2 (#ab3561), and goat antirabbit IgG FITC secondary antibody (#ab7086). All antibodies were purchased from Abcam. After excluding dead cells by gating, we measured live cells with NovoCyte (ACEA, San Diego, CA, USA). We analyzed them using NovoExpress software (ACEA) with 30,000 list mode events recorded for each sample by ruling out the background labeling with isotype controls (Köse et al., 2018).

### 2.5. Cell viability assay

We assessed the changes in LPS-stimulated (250 ng/mL, 1 day) LL24, LL29, and A549 cell proliferation on exposure to CBR agonists and/or antagonists on days 1, 3, and 5 using an MTT Cell Proliferation and Cytotoxicity Assay Kit (Boster Biological Technology, #AR1156, Pleasanton CA, USA). To examine the dose-dependent effects of CBR agonists and antagonists, 10^−3^, 10^−2^, or 10^−1^ mM of WIN55,212-2 or JWH015 w/wo 10^−2^ mM AM251 and AM630 were used. LPS or CBR agonist/antagonist untreated LL24, LL29, and A549 cells were used as control cells. We measured absorbance at 570 nm optical density by spectrophotometer (BMG Labtech, Ortenberg, Germany) (Önay et al., 2022). On day 5, half-maximal inhibitory concentration (IC_50_) and coefficient of determination (R^2^) values for CB1R agonist WIN55,212-2 and CB2R agonist JWH015 were calculated for LL24, LL29, and A549 cells using GraphPad Prism Version 10.1.2 (La Jolla, CA, USA).

### 2.6. Enzyme-linked immune sorbent (ELISA) assay

The levels of human TGF-β1 (#E-EL-0162), IL-8 (#E-EL-H6008), and TNF-α (#E-EL-H0109) were measured immunoenzymatically using commercially available kits (Elabscience, TX, USA) according to the manufacturer’s instructions in LL24, LL29, A549, and THP-1 cell supernatants. The levels of human ColI (BT Lab, #E1378Hu, Zhejiang, China) were measured immunoenzymatically according to the manufacturer’s instructions in LL24 and LL29 cell supernatants. LPS (250 ng/mL, 1 day) or CBR agonist/antagonist untreated LL24, LL29, A549, and THP-1 cell supernatants were used as controls. An optical density at 450 nm with a correction wavelength of 570 nm was determined for each ELISA sample using a microplate reader. TGF-β1, IL-8, TNF-α, and ColI concentrations were calculated using a standard curve (Aerts-Kaya et al., 2021). We calculated the half-maximal effective concentration (EC_50_) values for CB1R agonist WIN55,212-2 and CB2R agonist JWH015 for LL24, LL29, A549, and THP-1 cells using GraphPad Prism Version 10.1.2 (La Jolla, CA, USA) on day 5.

### 2.7. Statistical analysis

We performed the normality analysis of data using the Shapiro–Wilk test. The analysis of variance between multiple groups in repeated measures and Kruskal–Wallis tests were used as parametric and nonparametric tests, respectively, and a 95% confidence interval for statistical analysis was maintained. The descriptive statistics are presented as mean values ± SD and median, minimum, and maximum, according to the application of parametric or nonparametric tests, respectively. The results of the parametric data are presented with bar graphs to show the data distribution and error bars. The results of the nonparametric data are presented with box plots. The SPSS 23.0 Bivariate Correlation A (IBM Corp., Armonk, NY, USA) program was used for the analysis.

## 3. Results

### 3.1. Lung epithelial cells and fibroblasts present CB1R and CB2R

**Figure 2:**
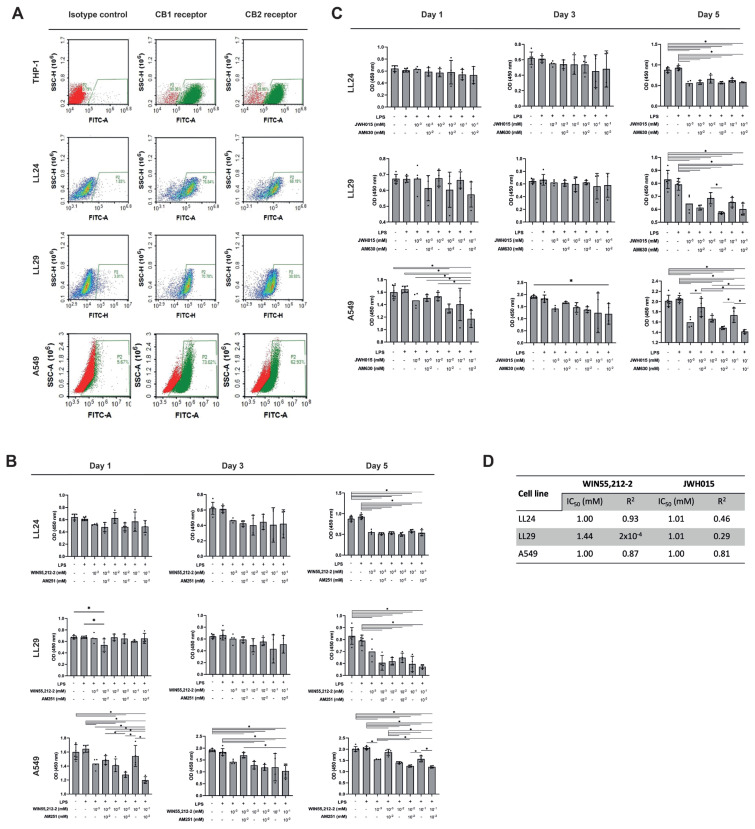
Lung fibroblast and epithelial cells express CB1R and CB2R, and CB1R and CB2R agonists have an antiproliferative effect on lung fibroblast and epithelial cells; (a) representative histograms quantitatively depicting CB1R and CB2R expression on the cell surface and inner membranes of THP-1, LL24, LL29, and A549 cells, as detected by FC, respectively; (b) MTT viability analysis in LL24, LL29, and A549 cells treated at 24 h with CB1R agonist WIN55,212-2 (10^−3^, 10^−2^, and 10^−1^ mM) only or with CB1R antagonist AM251 (10^−2^ mM) after 24-h LPS treatment. WIN55,212-2 inhibited cell growth in both cell types, but CBR independently on day 5 compared to LPS+ controls (*p < 0.05); (c) MTT viability analysis in LL24, LL29, and A549 cells treated at 24 h with CB2R agonist JWH015 (10^−3^, 10^−2^, and 10^−1^ mM) only or with CB2R antagonist AM630 (10^−2^ mM) after 24-h LPS treatment. CB2R agonist inhibited cell growth in both cell types CBR independently on day 5 compared to LPS+ controls (*p < 0.05); (d) IC_50_ (mM) and R^2^ values for WIN55,212-2 and JWH015 in LL24, LL29, and A549 cells (n = 3).

The CB1R expression of LL24, LL29, A549, and THP-1 cells was 76.84%, 70.70%, 73.02%, and 90.36%, respectively. The CB2R expression of LL24, LL29, A549, and THP-1 cells was 68.19%, 38.93%, 62.93%, and 89.96%, respectively. Figure 2a shows all results as a histogram.

### 3.2. CB1R and CB2R agonists inhibit lung fibroblast cell proliferation independently of CBRs and CBR agonist doses

WIN55,212-2 and JWH015 inhibited cell growth in LL24, LL29, and A549 cells at concentrations 10^−3^, 10^−2^, and 10^−1^ mM compared to WIN55,212-2 or JWH015 untreated controls on day 5 with and without LPS (p < 0.05) (Figures 2b and 2c). This inhibitory effect could not be reversed by AM251 or AM630 (both at 10^−2^ mM) (Figures 2b and 2c). When we treated high doses of WIN55,212-2 (10^−2^ and 10^−1^ mM) and AM251 (10^−2^ mM) together, this inhibited A549 cell growth compared to WIN55,212-2-treated A549 cells only on days 1 and 5 (p < 0.05) (Figure 2b). There was no difference in cell proliferation in LL24, LL29, and A549 cells with or without LPS (p > 0.05) (Figures 2b and 2c). The IC_50_ values for WIN55,212-2 were 1 mM in LL24 and A549 cells and 1.44 mM in LL29 cells. The IC_50_ values for JWH015 were 1.01 mM in LL24 and LL29 cells and 1 mM in A549 cells, measured on day 5 (Figure 2d).

### 3.3. CB1R and CB2R agonists have an antifibrotic effect independent of CB1R and CB2R

**Figure 3:**
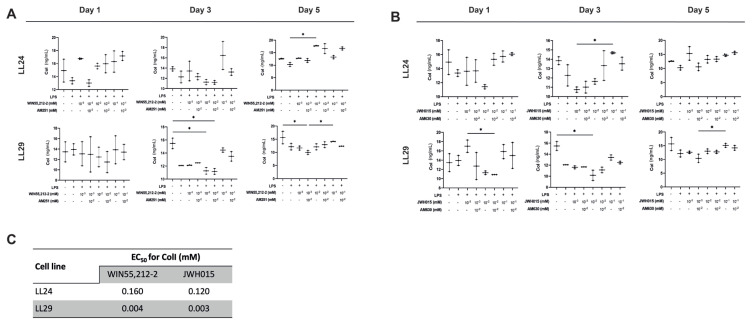
CB1R and CB2R agonists have an antifibrotic effect on lung fibroblast cells. Analysis of ColI levels in LL24 and LL29 cells treated at 24 h; (a) CB1R agonist WIN55,212-2 (10^−3^, 10^−2^, and 10^−1^ mM) and/or antagonist AM251 (10^−2^ mM) and (b) CB2R agonist JWH015 (10^−3^, 10^−2^, and 10^−1^) and/or antagonist AM630 (10^−2^) after 24-h LPS stimulation. LPS– cells were used as controls. Decreased ColI expressions were detected in LL29 treated with JWH015 and/or AM630 and with JWH015 on days 3 and 5 (n = 3) (*p < 0.05); (c) EC_50_ (mM) values for WIN55,212-2 and JWH015 in LL24, LL29, and A549 cells for ColI release (n = 3).

A significant difference was observed in the group treated with 10^−2^ mM WIN55,212-2 in LL24 cells compared to the LPS+ group on day 5 (p < 0.05) (Figure 3a). ColI expression decreased significantly in 10^−2^ mM WIN55,212-2 and/or AM251-treated and 10^−3^ mM WIN55,212-2-treated LL29 cells compared to the LPS– group on days 3 and 5, respectively (p < 0.05) (Figure 3a). JWH015 (10^−2^ mM) significantly decreased the ColI expression compared to the LPS– group in LL29 cells on day 3 (p < 0.05) (Figure 3b). LPS stimulation did not affect the ColI level in any groups (Figures 3a and 3b). The EC_50_ values of WIN55,212-2 and JWH015 for ColI in LL24 and LL29 cells are presented in Figure 3c.

### 3.4. CB1R and CB2R agonists increased TGF-β1 and TNF-α and decreased IL-8 cytokine levels dose-dependently

**Figure 4:**
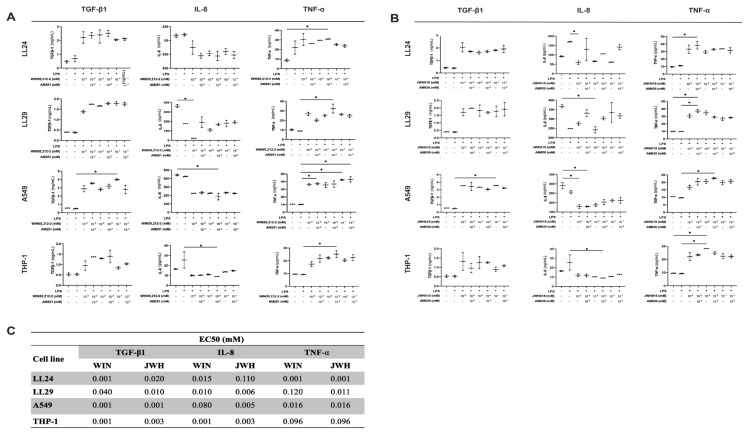
CBR agonists have a dose-dependent inflammation-modulation effect. Analysis of TGF-β1, IL-8, and TNF-α cytokine levels in LL24, LL29, A549, and THP-1 cell supernatants treated at 24 h with (a) CB1R agonist WIN55,212-2 (10^−3^, 10^−2^, and 10^−1^ mM) and/or antagonist AM251 (10^−2^ mM) and (b) CB2R agonist JWH015 (10^−3^, 10^−2^, and 10^−1^ mM) and/or antagonist AM630 (10^−2^ mM) after 24-h LPS stimulation. LPS– cells were used as controls. Elevated protein expressions of TGF-β1, TNF-α, and decreased protein expressions of IL-8 were detected in every cell type treated with WIN55,212-2 and/or AM251 and with JWH015 and/or AM630 at different concentrations in each cell type (n = 3) (*p < 0.05); (c) EC_50_ (mM) values for WIN55,212-2 and JWH015 in LL24, LL29, and A549 cells for TGF-β1, IL-8, and TNF-α release (n = 3).

TGF-β1 levels increased in LL24, LL29, and THP-1 cells treated with WIN55,212-2 and AM251 at all concentrations; however, this was not statistically significant (Figure 4a). Only 10^−1^ mM WIN55,212-2-treated A549 cells significantly increased TGF-β1 levels compared to the LPS+ group (p < 0.05). The IL-8 levels decreased in every cell treated with WIN55,212-2 and AM251 at all concentrations (Figure 4a). There was a significant decrease in the IL-8 levels in LL29 cells treated with 10^−3^ mM WIN55,212-2 and A549 cells treated with 10^−2^ mM WIN55,212-2 and AM251, compared to the LPS– controls (p < 0.05). Additionally, there was a significant decrease in the IL-8 levels in THP-1 cells treated with 10^−2^ mM WIN55,212-2 and AM251 compared to the LPS+ controls (p < 0.05). The TNF-α level significantly increased in LL24 cells treated with 10^−2^ mM WIN55,212-2 and AM251 compared to the LPS– group (p < 0.05). In addition, the TNF-α level increased in 10^−2^ mM WIN55,212-2 and AM251-treated LL29 cells, 10^−3^ mM and 10^−1^ mM WIN55,212-2 and AM251-treated A549 cells, and 10^−2^ mM WIN55,212-2 and AM251-treated THP-1 cells compared to the LPS+ controls (p < 0.05) (Figure 4a). LPS stimulation did not increase cytokine levels in any cells (Figure 4a).

In LL24, LL29, and THP-1 cells treated with JWH015 and AM630, the TGF-β1 levels increased at all concentrations; however, only 10^−1^ mM JWH015-treated A549 cells exhibited a significant increase in the TGF-β1 level compared to the LPS+ controls (p < 0.05) (Figure 4b). At all concentrations, the IL-8 level decreased in every cell type treated with JWH015 and AM630 (Figure 4b). A significant decrease was observed in the IL-8 levels in LL24 and THP-1 cells treated with 10^−3^ mM JWH015, 10^−2^ mM JWH015, and AM630 compared to the LPS+ controls, respectively (p < 0.05). In addition, there was a significant decrease in the IL-8 levels in LL29 cells treated with 10^−2^ mM JWH015 and A549 cells treated with 10^−3^ mM JWH015 and/or AM630 compared to the LPS– controls (p < 0.05). The TNF-α level increased in every cell type treated with JWH015 and AM630 at all concentrations (Figure 4b). The TNF-α level significantly increased in LL24 and LL29 cells treated with 10^−3^ mM JWH015 and AM630 and THP-1 cells treated with 10^−2^ mM JWH015 compared to the LPS– controls (p < 0.05). The IL-8 level increased in 10^−3^ mM JWH015 and AM630-treated LL29 cells, 10^−2^ mM JWH015 and AM630-treated A549 cells, and 10^−2^ mM JWH015-treated THP-1 cells compared to the LPS– controls (p < 0.05). LPS stimulation did not increase the TGF-β1, IL-8, and TNF-α levels in any of the cells (Figure 4b). Figure 4c presents the EC_50_ values of WIN55,212-2 and JWH015 for TGF-β1, IL-8, and TNF-α in LL24, LL29, A549, and THP-1 cells.

## 4. Discussion

This study showed that LL24, LL29, A549 and THP-1 cells express CB1R and CB2R. Our data is in line with previous studies investigating CB1 and CB2 mRNA and protein expression in A549 (Boyacioglu et al., 2021; Liu et al., 2022), human bronchial epithelial cells (Gkoumassi et al., 2007), peripheral blood (PB) monocytes (Montecucco et al., 2008), lung macrophages (Staiano et al., 2016), and mice lung tissue (Tahamtan et al., 2018; Huang et al., 2020). Although an increase in CB2R mRNA was observed in the lung tissue on day 21 in the bleomycin-induced IPF rat model, in our study, the expression rate of CB1R—especially CB2R—was found to be lower in LL29 than in LL24 (Liu et al., 2022). The majority of newly synthesized CBRs (70%) degraded rapidly by the first-order process (half-lives of approximately 5 h) (Howlett et al., 2010). For this reason, differences could be observed in the mRNA and protein levels of the receptors. In addition, immune cells like macrophages, which highly express CBRs, arrive in the lung after injury (Staiano et al., 2016). Therefore, as a result of the mRNA analysis performed on the entire lung tissue, it is possible that the CBR was higher than in the healthy tissue.

In this study, we found that both WIN55,212-2 and JWH015 inhibited LL24, LL29 and A549 cell proliferation on day 5. Similar to our results, CB1R agonists pirfenidone (1 mM) (Liu and Shi, 2019), CBD (10^−2^ mM) (Muthumalage and Rahman, 2019), CB2R agonists JWH015 (10^−2^ mM) (Liu and Shi, 2019), and JWH133 (10^−2^ mM) (Fu et al., 2017) significantly reduced the viability of human embryonic lung fibroblasts WI38 (Liu and Shi, 2019), mice lung fibroblasts mlg2908 (Fu et al., 2017), and HFL-1 cells (Muthumalage and Rahman, 2019) on day 2. These results, obtained using the same method and a similar dose range (10^−2^ mM) of CBR agonists as ours, prove that different CBR agonists also reduce the viability of lung fibroblasts. The fact that CB1R antagonist SR144528 (10^−3^ mM) inhibited this antiproliferative effect (Fu et al., 2017; Liu and Shi, 2019) but that AM251 and AM630 did not in our study suggest that it is due to AM251 and AM630 being inverse agonists or that the antiproliferative effect caused by WIN55,212-2 and JWH015 may be through other CBRs (Gkoumassi et al., 2007; Costola-de-Souza et al., 2013). Additionally, CB1R and CB2R agonist CBD (10^−2^ mM) significantly reduced the viability of bronchial epithelial cells BEAS-2B on day 2 (Muthumalage and Rahman, 2019). In our study, both WIN55,212-2 and JWH015 (10^−2^ mM) decreased the viability of A549 on days 1 and 3. Considering these results, we have shown that WIN55,212-2 and JWH015 (10^−2^ mM) may act as an efficient synthetic antiproliferative drugs candidates to reduce fibroblastic proliferation in IPF.

We found decreased ColI levels in WIN55,212-2 and JWH015-treated LL29 cells. This result is similar to that of previous research (Bronova et al., 2015) since the decrease was supported by CB1R and CB2R antagonists AM251 and AM630, respectively; thus, the regulation of agonist-mediated ColI release from fibroblasts may be through a different receptor than CB1R and/or CB2R. The CB2R agonist JWH133 (10^−2^ mM) inhibited TGF-β1-induced mice lung fibroblasts mlg2908 ColI mRNA expression (Fu et al., 2017). CB2R antagonist and inverse agonist SR144528 (10^−3^ mM) reversed the decrease in collagen mRNA (Fu et al., 2017). Considering our results, we determined that WIN55,212-2 and JWH015 (10^−2^ mM) reduced the proliferation and ColI production of lung fibroblasts through CBRs in vitro; thus, they could be candidates for in vivo studies as antifibrotic agents in IPF.

We demonstrated the inflammation-modulating properties of CBs by showing an increase in TGF-β1 and TNF-α levels, along with a decrease in IL-8 levels, in response to WIN55,212-2, JWH015, and their antagonists. These effects were observed to be dose- and CBR-independent across all cell types. CB2R agonists HU308 (10^−2^ mM) (Liu et al., 2020), pirfenidone (1 mM), and JWH015 (10^−2^ mM) (Liu and Shi, 2019) decreased the level of TNF-α of LPS-stimulated (1 μg/mL) mouse macrophage cells RAW264.7 (Liu et al., 2020) and lung-injured mouse BALF-stimulated human embryonic lung fibroblasts W138 (Liu and Shi, 2019) in cell supernatant on days 1 and 2. Similar to our results, this antiinflammatory effect was not inhibited by CB1R antagonist SR144528 (1 μM) (Liu and Shi, 2019). In our study, LPS (250 ng/mL) did not increase TNF-α in THP-1, LL24, and LL29 cells; on the contrary, a statistically significant increase in TNF-α was observed with WIN55,212-2 and JWH015 (10^−2^ mM). It may be that our LPS dose was insufficient. CB1R and CB2R agonists virodhamine and CP55,940 (both at 10^−2^ mM) significantly inhibited IL-8 release in TNF-α-stimulated (100 ng/mL) human bronchial epithelial cells (16HBE14o-) on day 1 (Gkoumassi et al., 2007). This inhibition was insensitive to CB1R antagonist SR141716A (1 mM), which is indirect evidence that this antiinflammatory response is mediated via CB2R. Additionally, CB2R antagonist SR144528 (1 mM) by itself reduced IL-8 release (Gkoumassi et al., 2007). Similarly, in our study, WIN55,212-2 and JWH015 reduced IL-8 release from A549 cells compared to LPS+ and LPS– controls; AM251 and AM630 did not affect IL-8 release. In line with our results, CB1R and CB2R activation by ACEA and JWH133, respectively (10^−5^ to 10^−2^ mM) did not change LPS-stimulated (1 μg/mL) production of TNF-α or IL-8 release in human lung macrophages and monocyte-derived macrophage culture supernatants compared to LPS-stimulated cytokine release by ELISA only (Staiano et al., 2016). In a similar study, the authors treated 10^−2^ mM CBD to normal primary human bronchial cells (NHBE), bronchial epithelial cells (BEAS-2B), monocytes derived from pleural effusion (U937), and lung fibroblasts (HFL-1) after being stimulated with LPS (1 μg/ml) and examined the release of TNF-α and IL-8 cytokines by ELISA (Muthumalage and Rahman, 2019). While monocytes stimulated with LPS released IL-8 to 4×10^3^ pg/mL and TNF-α to 35 pg/mL, when CBD was added, it reduced IL-8 to 2×10^2^ pg/mL and TNF-α to 2 pg/mL. Similarly, in our study, IL-8 decreased after applying 10^−2^ mM of WIN55,212-2 and JWH015 to LPS+ monocytes, while TNF-α increased. However, IL-8 in TNF-α-stimulated BEAS-2B cells treated with 10^−2^ CBD were similar to untreated cells. Similar to this result, although IL-8 decreased after applying 10^−2^ mM of WIN55,212-2 and JWH015 in LPS+ A549 in our study, it was not significant. When 10^−2^ mM CBD was applied, TNF-α released from LPS– U937, NHBE, and HFL-1 cells increased compared to CBD-untreated controls (Muthumalage and Rahman, 2019). Likewise, we observed that TNF-α and TGF-β1 increased with WIN55,212-2 and JWH015 despite LPS stimulation in the LL24 and LL29 cells. Because the agonists does were in line with previous studies (Gkoumassi et al., 2007; Staiano et al., 2016; Liu and Shi, 2019; Muthumalage and Rahman, 2019; Liu et al., 2020), it may seem that our LPS dose was insufficient. However, when the human primary dermal fibroblasts and epidermal keratinocytes were stimulated with a high dose of LPS (10 and 5 μg/mL, respectively) and JWH015 (10^−3^ mM), the amount of TNF-α increased significantly (Bort et al., 2017). While a decrease in TNF-α and TGF-β1 was detected in PB and lung tissue after CB application in in vivo studies (Costola-de-Souza et al., 2013; Liu et al., 2014a; Liu et al., 2014b; Liu and Shi, 2019; Liu et al., 2020; Mohammed et al., 2020; Parlar et al., 2021; Liu et al., 2022), no increase was observed in lung epithelium, fibroblasts, and monocytes (Staiano et al., 2016; Bort et al., 2017; Muthumalage and Rahman, 2019), suggesting that the inflammatory effect of these CBs originates from lung resident macrophages (Heukels et al., 2019; Liu et al., 2022). In addition, although LL24, LL29, A549, and THP-1 cells express CB1R and CB2R, they may have an antiinflammatory effect through other CBRs like TRPV1 and GPR55 (Gkoumassi et al., 2007; Costola-de-Souza et al., 2013).

CB1R agonists JWH133 (20 mg/kg, ip.) (Liu et al., 2014b), melilotus (25 mg/kg, oral) (Liu et al., 2014a), THC (20 mg/kg, ip.) (Mohammed et al., 2020), and CB2R agonist pirfenidone (300 mg/kg, oral) (Liu and Shi, 2019), AM1241 (3 mg/kg, ip.) (Parlar et al., 2021), YX-2102 (25 mg/kg, ip.) (Liu et al., 2022), HU308 (2.5 mg/kg, ip.) (Liu et al., 2020), JZL184 (16 mg/kg, ip.) (Costola-de-Souza et al., 2013) reduced TNF-α and TGF-β1 concentrations in BALF (Costola-de-Souza et al., 2013; Liu et al., 2014b; Mohammed et al., 2020), lung tissue (Liu et al., 2020), and PB serum (Liu et al., 2014a; Liu and Shi, 2019; Liu et al., 2022) in the bleomycin (Liu and Shi, 2019; Parlar et al., 2021; Liu et al., 2022), paraquat (Liu et al., 2014b), puncture (Liu et al., 2014a; Liu et al., 2020), endotoxin (Mohammed et al., 2020), and LPS-induced (Costola-de-Souza et al., 2013) lung injury mouse model at 2, 24 and 72 h compared to CB1R/CB2R agonist untreated controls, respectively. CB1R and CB2R antagonists AM281 (1 mg/kg) (Costola-de-Souza et al., 2013) and AM630 (5 mg/kg and 1 mg/kg) (Costola-de-Souza et al., 2013; Parlar et al., 2021) did not inhibit an increase in TNF-α on days 2 and 28, respectively. Similar to ours, these results show that the increase in TNF-α does not occur through classical CBRs.

The doses used for CBR agonists (10^−3^, 10^−2^, and 10^−1^ mM) and antagonists (10^−2^ mM) in this study are within the dose range in which we observed the physiological effects of CBs in our previous studies (Köse et al., 2018; Boyacioglu et al., 2021; Önay et al., 2022). This study determined the IC_50_ dose for WIN55,212-2 and JWH015 to be within the mM range, consistent with other in vitro studies examining CBR agonists. However, this dose is relatively high compared to the lower mM doses typically reported in in vivo studies. For example, Liu et al. (2020) and Liu and Shi (2019) reported antiinflammatory effects in vivo using much lower doses of CB2R agonists, such as HU308 and pirfenidone, at concentrations of approximately 2.5 mg/kg and 300 mg/kg, respectively. The discrepancy in effective dosing between in vitro and in vivo models could be attributed to several factors, including the complexity of biological systems in vivo, where drug bioavailability, metabolism, and tissue distribution play a significant role in modulating the physiological effects of these compounds. In contrast, in vitro systems lack these dynamic interactions and often require higher concentrations to achieve comparable effects. Moreover, while our in vitro results demonstrated a significant modulation of cytokine levels, translating these findings into a physiological context would require further investigation through in vivo studies to fine-tune the dosing and assess potential side effects. Thus, the higher IC_50_ dose observed in our study might not directly reflect therapeutic dosing in vivo. Still, it provides a foundational understanding that can guide future in vivo experiments to optimize dosage and minimize potential adverse effects.

The antiproliferative effects of WIN55,212-2 and JWH015 on LL24, LL29, and A549 cells may be attributed to several potential mechanisms. One possible pathway involves the inhibition of TGF-β1 signaling, known to play a crucial role in fibroblast activation and proliferation in pulmonary fibrosis. CB1R and CB2R activation have been linked to the downregulation of profibrotic pathways, including TGF-β1, through modulation of Smad-dependent signaling cascades (Fu et al., 2017). Furthermore, the antiinflammatory properties of WIN55,212-2 and JWH015 play a complementary role in their antiproliferative effects. CBR activation has been shown to inhibit NF-κB signaling, a crucial pathway involved in regulating proinflammatory cytokines, such as TNF-α and IL-8 (Mormina et al., 2006). By suppressing NF-κB, CBs can reduce inflammation, which is closely linked to fibrosis progression (Long et al., 2021). In particular, decreased levels of IL-8, observed in our study, indicate reduced neutrophil recruitment and inflammation, further contributing to the attenuation of fibroblast proliferation (Słoniecka et al., 2016). TNF-α, although increased in certain contexts, may also be part of a complex feedback mechanism regulating cell death and inflammation (Liu et al., 2020). These mechanistic insights suggest that CBs exert their antiproliferative effects through a combination of antifibrotic, proapoptotic, and antiinflammatory actions. By simultaneously targeting both fibrotic and inflammatory processes, WIN55,212-2 and JWH015 are promising as therapeutic agents in conditions like pulmonary fibrosis, where both pathways are critically involved in disease progression.

These preclinical study results are limited to an in vitro monolayer cell culture setting. Although the current design comprises healthy and IPF lung fibroblasts, lung epithelial cells, and PB monocytes, which meet all requirements for a complex in vitro setup, the findings should also be confirmed using in vivo animal models (Gkoumassi et al., 2007; Fu et al., 2017; Muthumalage and Rahman, 2019). Furthermore, the expression patterns of inflammation markers should be checked at the protein level using a large panel with different inflammatory agents or LPS doses. These limitations, however, do not constrain future in vivo and clinical studies because statistical accuracy was validated at the beginning of this study.

In conclusion, we determined that CB1R and CB2R agonists have inflammation-modulating and antifibrotic effects on normal human lung and IPF fibroblasts and human lung epithelial cells at specific concentrations. These results could be promising for in vivo studies and further clinical trials for the eventual reduction of the potential adverse effects of fibrosis and inflammation-targeted chemotherapeutics used for IPF in a clinical environment.
